# Epidemiology of prehospital emergency calls according to patient transport decision in a middle eastern emergency care environment: Retrospective cohort‐based

**DOI:** 10.1002/hsr2.2056

**Published:** 2024-04-23

**Authors:** Hassan Farhat, Guillaume Alinier, Kawther El Aifa, Ahmed Makhlouf, Padarath Gangaram, Ian Howland, Andre Jones, Cyrine Abid, Mohamed Chaker Khenissi, Ian Howard, Moncef Khadhraoui, Nicholas Castle, Loua Al Shaikh, James Laughton, Imed Gargouri

**Affiliations:** ^1^ Ambulance Service Hamad Medical Corporation Doha Qatar; ^2^ Faculty of Sciences University of Sfax Sfax Tunisia; ^3^ Faculty of Medicine ‘Ibn El Jazzar’ University of Sousse Sousse Tunisia; ^4^ University of Hertfordshire Hatfield UK; ^5^ Weill Cornell Medicine‐Qatar Doha Qatar; ^6^ Northumbria University Newcastle upon Tyne UK; ^7^ College of Engineering Qatar University Doha Qatar; ^8^ Faculty of Health Sciences Durban University of Technology Durban South Africa; ^9^ Laboratory of Screening Cellular and Molecular Process, Centre of Biotechnology of Sfax University of Sfax Sfax Tunisia; ^10^ Higher Institute of Biotechnology University of Sfax Sfax Tunisia; ^11^ Faculty of Medicine University of Sfax Sfax Tunisia

**Keywords:** cohort study, emergency medical service, Middle East, patient decisions, prehospital care

## Abstract

**Background and Aim:**

Though emergency medical services (EMS) respond to all types of emergency calls, they do not always result in the patient being transported to the hospital. This study aimed to explore the determinants influencing emergency call‐response‐based conveyance decisions in a Middle Eastern ambulance service.

**Methods:**

This retrospective quantitative analysis of 93,712 emergency calls to the Hamad Medical Corporation Ambulance Service (HMCAS) between January 1 and May 31, 2023, obtained from the HMCAS electronic system, was analyzed to determine pertinent variables. Sociodemographic, emergency dispatch‐related, clinical, and miscellaneous predictors were analyzed. Descriptive, bivariate, ridge logistic regression, and combination analyses were evaluated.

**Results:**

23.95% (*N* = 21,194) and 76.05% (*N* = 67,285) resulted in patient nontransport and transportation, respectively. Sociodemographic analysis revealed that males predominantly activated EMS resources, and 60% of males (*n* = 12,687) were not transported, whilst 65% of females (*n* = 44,053) were transported. South Asians represented a significant proportion of the transported patients (36%, *n* = 24,007). “Home” emerged as the primary emergency location (56%, *n* = 37,725). Bivariate analysis revealed significant associations across several variables, though multicollinearity was identified as a challenge. Ridge regression analysis underscored the role of certain predictors, such as missing provisional diagnoses, in transportation decisions. The upset plot shows that hypertension and diabetes mellitus were the most common combinations in both groups.

**Conclusions:**

This study highlights the nuanced complexities governing conveyance decisions. By unveiling patterns such as male predominance, which reflects Qatar's expatriate population, and specific temporal EMS activity peaks, this study accentuates the importance of holistic patient assessment that transcends medical histories.

## 1 INTRODUCTION

Emergency Medical Services (EMS) constitute a fundamental cornerstone of prehospital care to guarantee prompt medical interventions for patients outside traditional healthcare settings. EMS ensures timely patient transfer to dedicated facilities for comprehensive medical examination and treatment. The continuous development of EMS has mirrored the dynamic necessities of a populational requirement for assured, efficacious emergency care.^1^ A competitive prehospital care system is an important indicator of effective patient outcomes.^2^

To ensure service excellence, EMS have instituted dedicated emergency helplines (e.g., 999, 911, and 190) in some Middle Eastern and North African countries and indicated their unwavering allegiance to public health and safety.^3^ Various patients refuse transportation to healthcare institutions after emergency response and onsite medical care provision.^4^ This behavioral conundrum impacts EMS efficiency and judicious resource allocation, with broader ramifications for patient health outcomes that necessitate the identification of epidemiological decision‐making‐related factors.

Qatar, similar to its Middle Eastern counterparts, presents a rich tapestry of demographics, with male‐dominated demographic configurations predominantly populated by South Asians and Arabs, including indigenous Qataris.^5^ Qatar's leading prehospital emergency medical care provider is the Hamad Medical Corporation Ambulance Service (HMCAS) stands as the sole provider in the country, ensuring emergency medical responses for the community through the 999 emergency call service.^6^ The emergency response units (ERU) are distributed across eight hubs and locations where paramedics commence their shifts, replenish their response units, and respond to all emergency calls to the HMCAS communication call centre in the National Command Center (NCC).^3^ On receiving a call, operators identify a medical emergency and transfer the call to the HMCAS emergency medical dispatchers (EMD) for processing and triage using the computer‐aided ProQA™ dispatch system.^7^ The EMD then dispatches the most appropriate ERU and provides emergency callers prehospital safety and lifesaving instructions until the ERU arrives. The ERU crew provides appropriate emergency medical assessment and treatment if needed, according to their HMCAS Clinical Practice Guidelines (CPG)‐defined scope of practice.^8^ In Qatar, patients or their legal guardians can refuse transportation to the hospital by signing the electronic patient report form (ePCR). The HMCAS operational ethos gravitates towards encouraging patient conveyance to hospitals rather than primary healthcare centers and does not involve clinician‐advised non‐conveyance, given the risk of undertriaging due to language barriers or unusual critical clinical presentations that require in‐hospital diagnostic intervention and clinical care. The HMCAS ERU consist of Alpha, Bravo, Charlie, and Delta units. Alpha and Bravo have two Ambulance Paramedics (AP) competent in conducting emergency medical evaluations and administering emergency treatment. Charlie's units consist of a Critical Care Paramedic and Assistant equipped for more advanced interventions. Delta units, led by a senior supervisor, manage multi‐agency scenes.

The complex epidemiological framework guiding patient‐conveyance decisions in the Middle East remains under‐explored. A granular analysis of potential determinants will enable judicious strategies and informed decision‐making. We posited that an amalgamation of human sociodemographic, clinical, and potentially systemic factors contributes to conveyance determination in Qatar. This study aimed to outline the various determinants influencing patients' conveyance decisions following prehospital emergency calls in the Middle Eastern environment.

## 2 METHODS

### 2.1 Study design and setting

This retrospective quantitative cohort (Transported vs. Not Transported) analysis of 93,712 emergency calls received between January 1 and May 2023 involved data from the HMCAS electronic record system managed by the business intelligence (BI) division. This study adhered to the Consolidated Standards of Reporting Trials (CONSORT) guidelines for cohort studies and was approved by the HMC Medical Research Center (Reference: MRC‐01‐22‐264). We used R‐Studio™ for data arrangement and analyses.

### 2.2 Participants

The inclusion criterion was 999 emergency calls that resulted in at least one ERU dispatch wherein the paramedic performed onsite patient assessment, with either hospital conveyance or a patient decision against it. The exclusion criteria were: (1) cases involving a deceased patient and (2) calls originating from healthcare facilities, as patients can still receive timely medical attention, offsetting the need for advanced care, unlike that in community‐based 999 emergency callers.

### 2.3 Variables

The HMCAS BI team provided an initial data set comprising 73 variables. After data wrangling, six variables underwent nomenclature adjustments, 14 new variables were derived or transformed from their preliminary configurations, and 47 were excluded. Two supplementary variables, namely the longitude and latitude of the 999 calls, were incorporated to construct the emergency call map. Twenty variables were retained for in‐depth analysis and classified as outcome variables and sociodemographic, EMD‐related, clinical, and miscellaneous predictors.

#### 2.3.1 Outcome variable

The variable “Handover” was designated as the outcome variable to segment the cohort into “Transported” and “Not Transported” groups as follows:

i. “Transported” group: Patients who were conveyed to hospitals following a 999 call and an on‐scene assessment by the HMCAS crew.
ii. “Not Transported” group: Encompassed three sub‐categories: “Refused Transport ‐ Treated At Scene,” “Refused Transport,” and “Treated At Scene ‐ Not Transported.”

Entries labeled “DOA (death on arrival) Not Transported” were systematically omitted from the analysis.

#### 2.3.2 Sociodemographic predictors

The categorical variables were: (1) sex, (2) nationalities represented as “Nationalities_CAT,” (3) age categorized as “Age_CAT,” (4) region, and (5) weight categorized as “Weight_CAT.” Categorization of age and weight is a common practice in the clinical field and helps provide a more nuanced understanding of risk factors across different subgroups.^9^

#### 2.3.3 Emergency medical dispatch‐related predictors

i. The Categorical Variables were: (1) Call Service Owner denoted as “CFS_Owner,” (2) emergency caller's geographical coordinates represented as “Location_LAT” and “Location_Long,” (3) type of location denoted as “LocationType,” 4) ProQA™ Protocol Labeled as “ProtocolName,” (5) dispatch type: defined as “DispatchType,” (6) response priority levels for the scene (“PriorityToScene”) and hospital (“PriorityToHospital”), and (7) ERU type denoted as “Unit_Type.”
ii. The Continuous Variables were: (1) Unit identification time in minutes from the 999 call until the nearest unit is identified, referred to as “TimeToFindTheNearestUnit.” (2) Response Duration was defined as the time the ERU took to reach the scene, labeled “TimeToReachOnScene.” (3) Patient Interaction Duration: Span from the paramedic's arrival to either the patient handover to a healthcare facility or obtaining a refusal form signature, referred to as “TimeWithPatientUntilAvailable.” (4) Unit in‐Dispatch duration: Duration from the dispatch of the ERU until it is available for the next call, denoted as “TimeFromDispatchUntilAvailable.”

#### 2.3.4 Clinical predictors

The clinical predictors are all categorical and include (1) Provisional diagnoses categorized as “ProvisonalDiagnoses_CAT,” (2) Receiving facility denoted “TransportedTo,” (3) Receiving unit referred to as “PatientTriagedArea.” The comorbidities were each under a separate variable, including (1) Pregnancy “CurrentlyPregnant,” (2) Asthma, (3) Cardio‐Artery‐Disease “CAD,” (4) Chronic Obstructive Pulmonary Disease “COPD,” (5) Cardio‐vascular Accident “CVA,” (6) Seizure, (7) Diabetes Mellitus “DM,” (8) Hypertension, (9) Surgeries, (10) Others, (11) None, and (12) Unknown.

#### 2.3.5 Miscellaneous predictors

i. Categorical: (1) Week of the year “WeekNumber,” (2) Day of the week “Week Day.”
ii. Continuous: (3) Hour of the day when an emergency call was received, referred to as “Hour_Received.”

### 2.4 Statistical methods

Descriptive statistical analyses were performed by calculating the count and percentage of categorical variables and the median for continuous variables. Shewhart Statistical Process Control (SPC) charts were designed to observe the time‐series variations in transported patients following 999 emergency calls. A function for bivariate analysis was created in R (Supporting Information S1: Appendix 1 and Appendix 2). The null hypothesis (H_0_) was: “*There is no correlation between Handover and the studied categorical variables*.” The chi‐square test for categorical variables determined a significant association between two categorical variables. For specific variables that retained categories with low counts after exhaustive iterations where the chi‐square test was unsuitable because of data sparsity, the variables were refined. Fisher's exact test was used as needed. Cramer's V coefficient was used to measure the strength of the association of categorical predictors with transport groups (range: 0 [indicating no association] to 1 [indicating perfect association]).^10^ Odds ratios (OR), which measure the exposure‐outcome association and indicate the intergroup odds of an event happening, were calculated. For continuous variables, mean intergroup differences were determined using the Mann–Whitney *U* test.^11^ Ridge regression, used to handle multicollinearity, was used,^12^ and the outcome variable ‘Handover’ was transformed into a categorical format to facilitate binary logistic regression.^13^ The ridge regression model facilitated the extraction of coefficients indicating the influence probabilities of each predictor on “Handover.” Comorbidity is crucial in patient management and prognosis.^14^, ^15^ A comorbidity combination analysis was conducted by creating UpSet plots, a visualization technique to depict more than three intersecting sets,^16^, ^17^ It enabled a greater understanding of the interaction and confluence of different comorbidities on patient‐conveyance decisions.

## 3 RESULTS

### 3.1 Descriptive statistics

**Figure 1:**
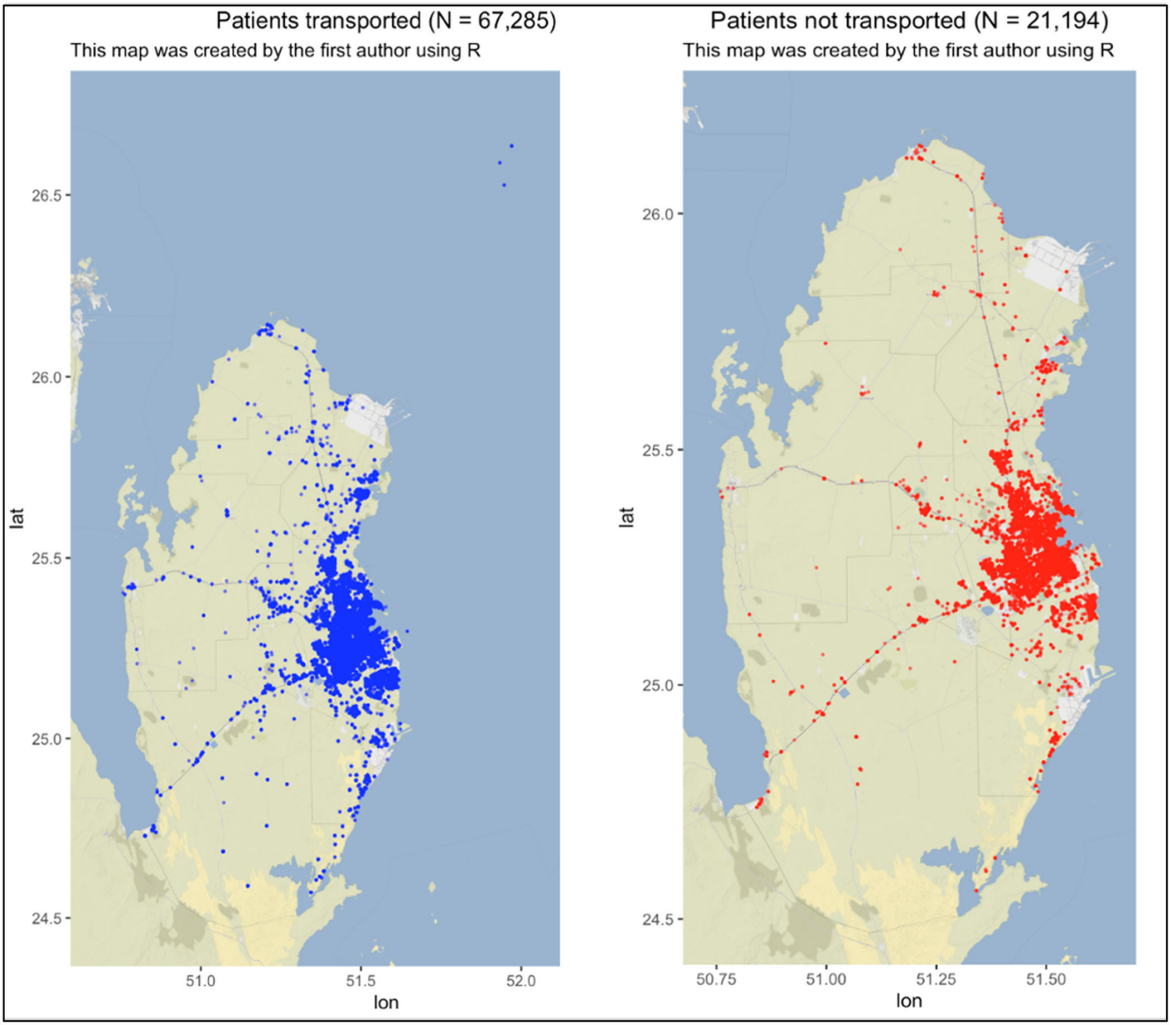
Map for distribution of patients transported and not transported by the HMCAS. *(The dimensions of the map are determined automatically by the ‘ggmap’ package according to the coordinates provided)*

Among the 88,479 participants enrolled after data wrangling (Figure 1), 67,285 (76.05%) and 21,194 (23.95%) were and were not transported, respectively. Supporting Information S1: Appendix 3 shows unstable weekly variations in the number of transported patients, which increased interpretation‐related challenges, whilst the intraday variation showed increasing proportions of transported patients from 9 a.m. to 12 p.m. that significantly decreased in the evening and early morning.

Table 1 presents descriptive data on the study population. The sociodemographic predictors showed a predominantly male representation in both the transported (65%; 44,053) and non‐transported (60%; 12,687) groups. Within nationalities, South Asians constituted the largest portion of the transported group (36%; 24,007), whereas the non‐transported group exhibited roughly equivalent proportions of Qataris, Middle East, North Africa (MENA), and South Asians. Approximately half of the individuals in both categories resided in urban areas (Table 1 and Figure 1). The age demographic most represented across both groups was 29–44 years. A significant proportion of both groups weighed 70–95 kg.

**Table 1:** Descriptive statistics results.

Characteristic	Not transported	Transported
	*N* = 21,194^*1*^	*N* = 67,285^*1*^
1. SOCIODEMOGRAPHIC PREDICTORS		
Gender		
Female	8505 (40%)	23,209 (34%)
Male	12,687 (60%)	44,053 (65%)
Missing	2 (<0.1%)	23 (<0.1%)
Nationalities_CAT		
Qatari	5094 (24%)	11,187 (17%)
GCC Other	893 (4.2%)	2513 (3.7%)
MENA	5,363 (25%)	14,463 (21%)
East Asia & Pacific	968 (4.6%)	3699 (5.5%)
South Asia	5052 (24%)	24,007 (36%)
Sub‐Saharan Africa	1899 (9.0%)	6848 (10%)
Europe and Central Asia	936 (4.4%)	1565 (2.3%)
North America	139 (0.7%)	244 (0.4%)
Latin America & Caribbean	45 (0.2%)	90 (0.1%)
Other	495 (2.3%)	1257 (1.9%)
Missing	310 (1.5%)	1412 (2.1%)
Region		
Urban	9667 (46%)	31,361 (47%)
Rural	3441 (16%)	12,705 (19%)
Missing	8086 (38.1%)	23,219 (34.1%)
Age categories		
Age<14	2488 (12%)	7459 (11%)
14≤Age<29	6021 (28%)	17,669 (26%)
29≤Age<44	7314 (35%)	24,273 (36%)
44≤Age<59	2698 (13%)	9894 (15%)
59≤Age<75	1720 (8.1%)	5167 (7.7%)
75≤Age<90	845 (4.0%)	2300 (3.4%)
Age≥90	108 (0.5%)	353 (0.5%)
Missing	0 (0%)	170 (0.3%)
Weight_CAT		
Weight<45	2273 (11%)	6833 (10%)
45≤Weight<70	8609 (41%)	24,513 (36%)
70≤Weight<95	9159 (43%)	31,244 (46%)
95≤Weight<120	995 (4.7%)	4018 (6.0%)
Weight≥120	154 (0.7%)	629 (0.9%)
Missing	4 (<0.1%)	48 (<0.1%)
2. EMERGENCY MEDICAL DISPATCH‐RELATED PREDICTORS		
CFS owner		
EMS	10,096 (48%)	34,499 (51%)
No call taking/missing	8084 (38%)	23,193 (34%)
Other	3014 (14%)	9,593 (14%)
Dispatch type		
Zulu (Z)	2611 (12%)	11,309 (17%)
Yankee (Y)	6285 (30%)	24,338 (36%)
Xray (X)	4170 (20%)	8337 (12%)
Tango (T)	17 (<0.1%)	23 (<0.1%)
Uncompleted ProQa	26 (0.1%)	85 (0.1%)
No call taking/Missing	8084 (38%)	23,193 (34%)
Not in use	1 (<0.1%)	0 (0%)
Location type		
Airport	3266 (15%)	2433 (3.6%)
Beach/sea/ocean	85 (0.4%)	296 (0.4%)
Farm	36 (0.2%)	232 (0.3%)
Home	10,259 (48%)	37,725 (56%)
Industrial area	118 (0.6%)	1589 (2.4%)
Other	946 (4.5%)	2175 (3.2%)
Public area	737 (3.5%)	2,119 (3.1%)
Recreation (sport)	52 (0.2%)	252 (0.4%)
School	353 (1.7%)	1222 (1.8%)
Street (road)	4206 (20%)	13,762 (20%)
Work	523 (2.5%)	3941 (5.9%)
Missing	613 (2.9%)	1539 (2.3%)
Priority to scene		
P1	17,411 (82%)	56,595 (84%)
P2	3459 (16%)	10,382 (15%)
Missing	324 (1.5%)	308 (0.5%)
Priority to hospital		
P1	0 (0%)	3367 (5.0%)
P2	0 (0%)	62,418 (93%)
P3	0 (0%)	436 (0.6%)
Missing	0 (0%)	1064 (1.6%)
ERU Type		
Alpha	8453 (40%)	29,612 (44%)
Bravo	1425 (6.7%)	641 (1.0%)
Charlie	996 (4.7%)	4724 (7.0%)
Delta	1157 (5.5%)	3730 (5.5%)
Hazmat	285 (1.3%)	1488 (2.2%)
Life Flight (LF)	72 (0.3%)	543 (0.8%)
Specialized Emergency Management	322 (1.5%)	1202 (1.8%)
Other	397 (1.9%)	2152 (3.2%)
Missing	8087 (38%)	23,193 (34%)
3. CLINICAL PREDICTORS		
Transported to		
Airport clinics	0 (0%)	1642 (2.4%)
Governmental with no prenotification	0 (0%)	58,064 (86%)
Governmental with prenotification	0 (0%)	331 (0.5%)
Pediatric Emergency Care (pec)	0 (0%)	5763 (8.6%)
Private	0 (0%)	457 (0.7%)
Other	0 (0%)	144 (0.2%)
Patient triaged area		
Adult assessment ED	0 (0%)	36,325 (54%)
Low acuity ED	0 (0%)	16,713 (25%)
Bypass criteria ED	0 (0%)	3703 (5.5%)
Obstetrics/gynecology ED	0 (0%)	2426 (3.6%)
Pediatric ED	0 (0%)	5589 (8.3%)
Dialysis	0 (0%)	2 (<0.1%)
Other	0 (0%)	1037 (1.5%)
Comorbidities		
Asthma	777 (3.7%)	2368 (3.5%)
CAD	750 (3.5%)	2996 (4.5%)
COPD	64 (0.3%)	261 (0.4%)
Cva	140 (0.7%)	911 (1.4%)
Seizure	170 (0.8%)	1096 (1.6%)
DM	2678 (13%)	9093 (14%)
Hypertension	2768 (13%)	9953 (15%)
None	13,892 (66%)	40,072 (60%)
Others	2285 (11%)	9533 (14%)
Surgeries	360 (1.7%)	1797 (2.7%)
Unknown	810 (3.8%)	4489 (6.7%)
Currently pregnant	306 (1.4%)	2884 (4.3%)
4. MISCELLANEOUS PREDICTORS		
Weekday		
Sunday	1644 (7.8%)	5686 (8.5%)
Monday	2135 (10%)	7960 (12%)
Tuesday	1748 (8.2%)	6787 (10%)
Wednesday	2207 (10%)	7088 (11%)
Thursday	1798 (8.5%)	5248 (7.8%)
Friday	1725 (8.1%)	5487 (8.2%)
Saturday	1853 (8.7%)	5836 (8.7%)
Missing	8084 (38%)	23,193 (34%)
^*1*^ *n* (%); Median (IQR)		

**Figure 2:**
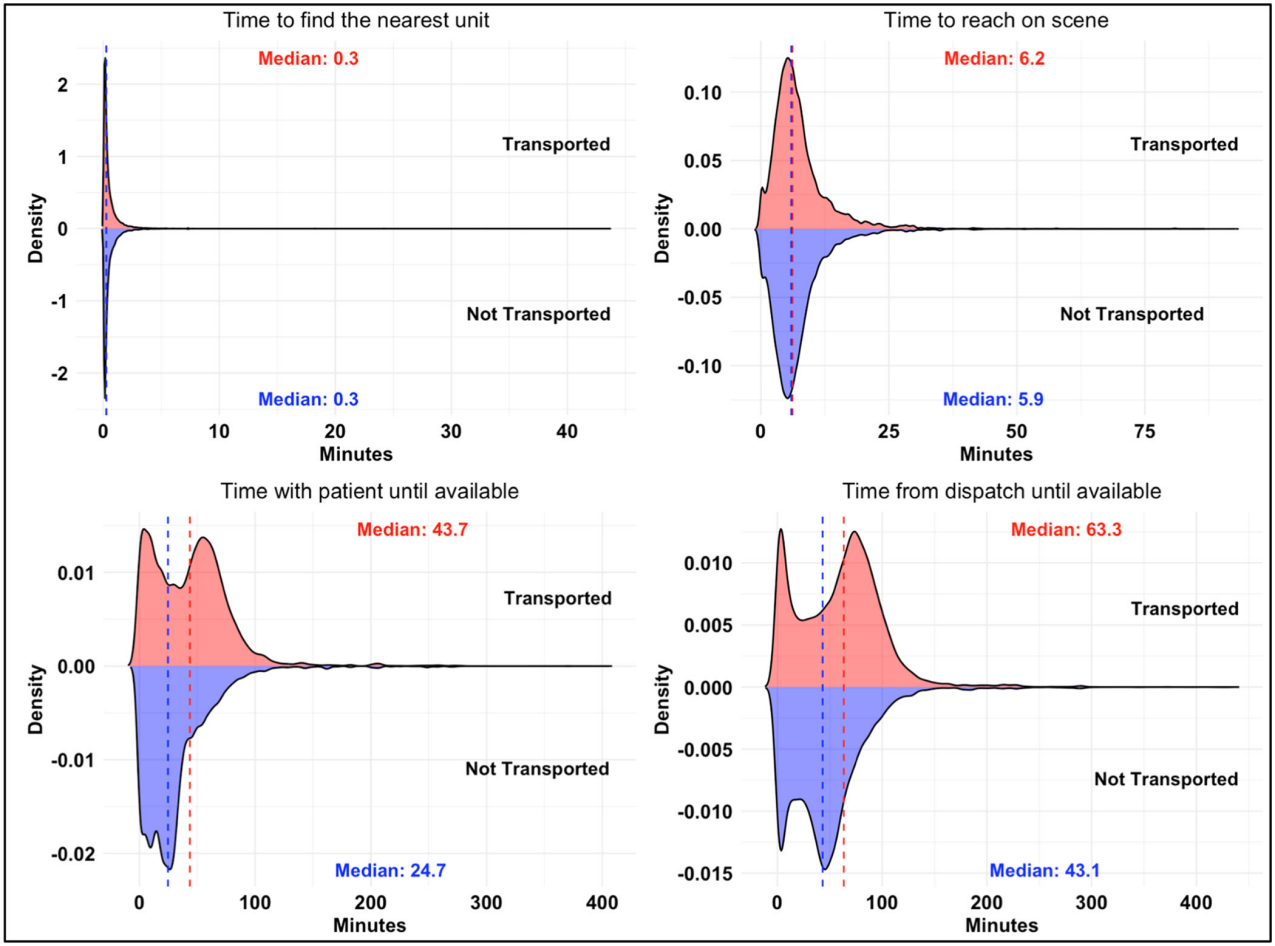
Mirror plots of dispatch and response durations distribution.

The median response times were 6.2 and 5.9 min (within the international benchmark) (Figure 2), and the time from ERU dispatch until assigned was 63.3 and 43.1 min for transported and non‐transported patients, respectively. The Yankee (Y) and “No call taking/Missing” dispatch type was predominant among transported and non‐transported patients, respectively, which includes walk‐in patients who visit nearby HMCAS ERU standby points instead of calling 999 because of location proximity. As expected, the primary emergency location was “Homes” for both categories because 999 emergency calls are community‐generated. In both groups, the majority had a “P1” priority to the scene where ambulances moved with lights and sirens.^3^ Alpha was the predominant ERU category. For the ProQA™ call‐taking protocols, RTA (P29) and sick persons (P26) were the predominant protocols used in both groups (Supporting Information S1: Appendix 4). For clinical variables, patients were predominantly transported to governmental healthcare facilities without prenotification requirements. Most patients were triaged at an adult assessment Emergency Department (ED), as they did not require critical care. Despite several comorbidities, a significant percentage of patients in both groups presented without known comorbidities, with the predominant category of a provisional diagnosis of low‐acuity trauma and medical care (Supporting Information S1: Appendix 4). Considering miscellaneous predictors, despite considerable missing data, Monday was the peak day for prehospital 999 emergencies in the transported group, whereas the non‐transported group saw a weekend surge (Table 1).

### 3.2 Bivariate and multivariate analyses

Table 2 presents the association level of both cohort groups with the remaining variables. Most variables showed significant associations between both groups and continuous and categorical variables (*p* < 0.01). The strength of these associations could be inferred from the Cramer's V values; for instance, “ProtocolName” has a moderate association strength of 0.15, while “LocationType” has a stronger association at 0.22. It is worth noting, however, that some conditions like “Asthma” and “COPD” were not significantly associated with both “Handover” groups. Variables with an OR greater than one had a greater likelihood of the “Handover” event not being in the Not Transported group. An undetermined OR indicates a potentially strong but nonquantifiable association warranting large‐sample investigation. For the Mann‐Whitney‐U tests, significant differences in distributions were observed for “Hour_Received,” “TimeToReachOnScene,” “TimeWithPatientUntilAvailable,” and “TimeFromDispatchUntilAvailable” (*p* < 0.01). However, “TimeToFindTheNearestUnit” did not show a statistically significant difference. The significant association in the bivariate analysis with most variables indicate that many examined variables were significantly associated or showed intergroup differences, suggesting potential multicollinearity, which could distort the estimated regression coefficients.

**Table 2:** Bivariate analysis.

1) Summary of chi‐square tests for categorical variables								
Variable	Chi‐Square statistic	Degrees of freedom		*p*‐Value		Cramer's V	Odds ratio	
CFS_Owner	103.82	3		<0.01		—	—	
ProtocolName	1915.60	33		<0.01		0.15	—	
DispatchType	1081.50	6		<0.01		0.04	—	
PriorityToScene	274.40	2		<0.01		0.06	—	
PriorityToHospital	88,479	4		<0.01		1	—	
Week Day	180.78	7		<0.01		0.04	—	
Region	131.18	3		<0.01		0.04	—	
LocationType	4,422.80	11		<0.01		0.22	—	
Hour_Received	455.86	23		<0.01		0.07	—	
Gender	225.43	2		<0.01		0.05	—	
Nationalities_CAT	1672.55	10		<0.01		0.14		
Age_CAT	161.36	7		<0.01		0.05	—	
Weight_CAT	179.03	5		<0.01		0.05	—	
Unit_Type	2,775.06	8		<0.01		0.17	—	
WeekNumber	163.14	14		<0.01		0.04	—	
Asthma	0.97	1		0.32		0.00	0.96	
CAD	32.98	1		<0.01		0.02	1.27	
COPD	3.02	1		0.08		0.00	1.29	
CVA	65.43	1		<0.01		0.03	2.06	
Seizure	77.53	1		<0.01		0.03	2.04	
DM	10.71	1		<0.01		0.01	1.08	
Hypertension	39.135	1		<0.01		0.02	1.15	
None	242.91	1		<0.01		0.05	0.77	
Others	159.45	1		<0.01		0.04	1.36	
Surgeries	63.63	1		<0.01		0.03	1.59	
Unknown	231.97	1		<0.01		0.05	1.78	
CurrentlyPregnant	724.17	2		<0.01		‐	‐	

2) Summary of Mann–Whitney‐*U* tests for continuous variables								
Variable	**Statistic**		***p*‐Value**		**CI_lower**			**CI_upper**
Hour_Received	730,904,066		<0.01		4.12 × 10^−08^			4.66 × 10^−07^
TimeToFindTheNearestUnit	713,927,809		0.77		−6.15 × 10^−08^			1.22 × 10^−08^
TimeToReachOnScene	678,944,256		<0.01		−0.35			−0.25
TimeWithPatientUntilAvailable	527,265,766		<0.01		−13.44			−12.48
TimeFromDispatchUntilAvailable	544,683,551		<0.01		−1.57			−1.47

### 3.3 Ridge logistic regression analysis

**Figure 3:**
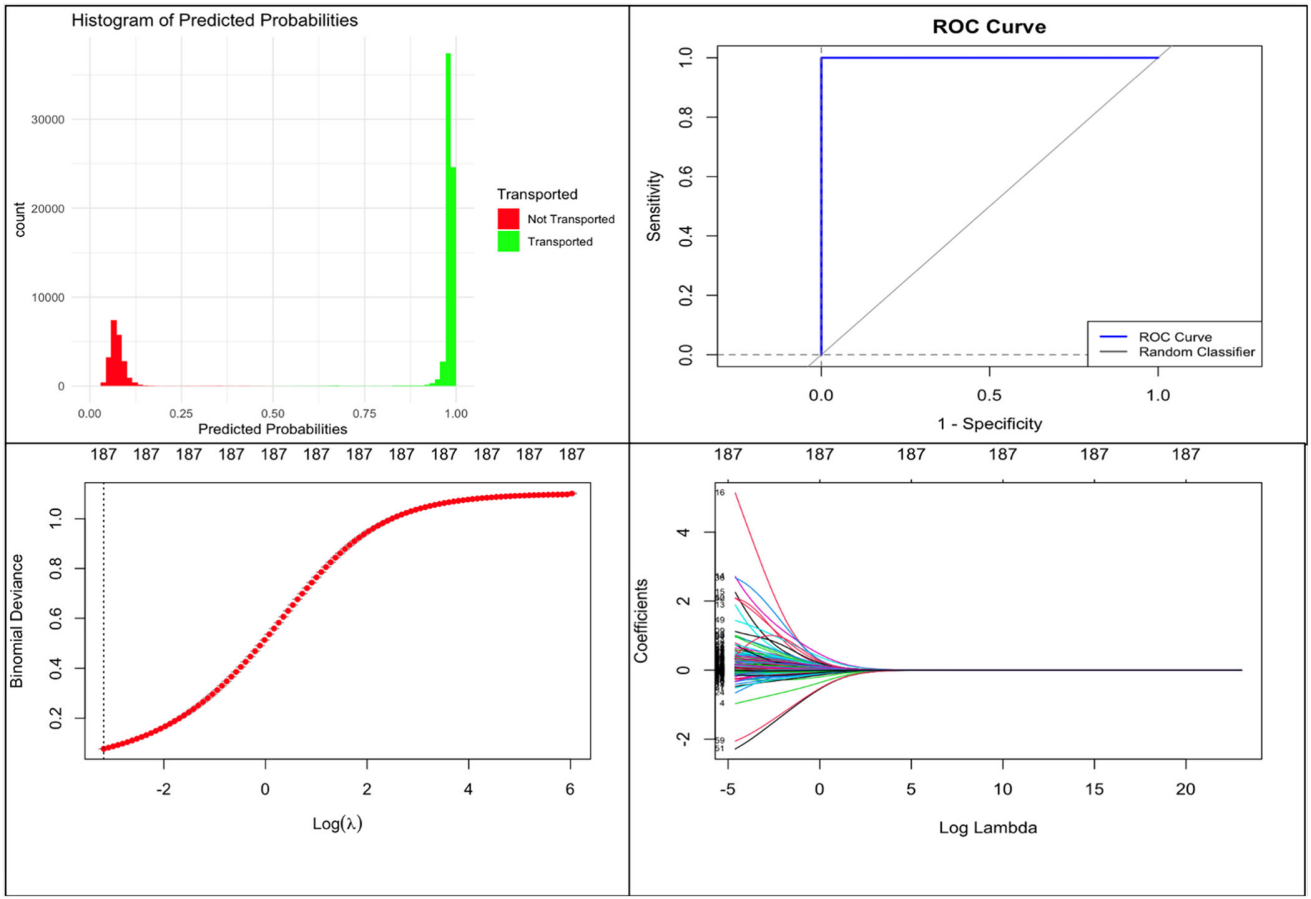
Plots of the results of the ridge regression analysis.

To address potential issues related to multicollinearity, ridge regression was applied to our data set (Table 3 and Figure 3) after encoding the categorical variables and preventing the regression model from being overly influenced by correlated predictors to ensure more reliable findings. Figure 5 included the Lambda plots, histogram for predicted probabilities, and the receiver operating characteristic area under the curve (ROC AUC) plot. The Lambda plots enable minimizing the model's generalization error, resulting in more robust results. The histogram for predicted probabilities provides insights into the model's calibration, specifically how well the predicted probabilities align with the observed outcomes. Notably, a significant number of predicted probabilities cluster around a value of 1, coherent with the findings presented in the ROC AUC and Table 3 and indicative of a high likelihood for certain cases to be transported. The ROC AUC is a graphical representation that illustrates the diagnostic ability of our logistic ridge regression model at varying classification thresholds. It ranges between zero and one. The closer to 1, the better. The coefficients in Table 3 indicated a high likelihood for certain cases to be transported. Positive coefficients indicated that the chances of a ‘Transported’ outcome increased as certain predictors increased. For example, patients with missing provisional diagnoses are more likely to be transported. Cases diagnosed with “Cardiac Arrest” were more likely to be transported as expected, while a “Hypoglycemia” diagnosis tended to decrease transportation likelihood.

**Table 3:** Ridge regression coefficients analysis results.

Variable	Coefficients
PriorityToHospitalMissing	3.08
ProvisonalDiagnoses_CATMissing	2.11
TransportedToNot Applicable	−1.78
PriorityToHospitalP2	1.70
PatientTriagedAreaNot Applicable	−1.65
GenderMissing	1.55
TransportedToOther	1.48
TransportedToGouvernemental no Prenotif	1.16
PriorityToHospitalP3	1.11
Weight_CATMissing	0.97
ProvisonalDiagnoses_CATDOA	0.95
PriorityToHospitalP1	0.92
TransportedToPrivate	0.74
TransportedToPEC	0.70
ProvisonalDiagnoses_CATCardiac Arrest	0.64
ProtocolName9	0.54
ProtocolName31	−0.54
TransportedToGouvernmental with Prenotif	0.54
PatientTriagedAreaDialysis	0.48
PatientTriagedAreaLow_Acuity_ED	0.48
PatientTriagedAreaPaed_ED	0.47
ProvisonalDiagnoses_CATCOPD	0.47
ProvisonalDiagnoses_CATCardiovas_Other	0.42
ProvisonalDiagnoses_CATHemothorax	0.41
LocationTypeBeach/Sea/Ocean	0.40
PatientTriagedAreaByPass_Crit_ED	0.39
ProvisonalDiagnoses_CATCroup/Epiglottitis	0.38
ProtocolName15	0.37
ProvisonalDiagnoses_CATHypoglycemia	−0.37
ProvisonalDiagnoses_CATPneumothorax	0.37

### 3.4 Comorbidities combination analysis

**Figure 4:**
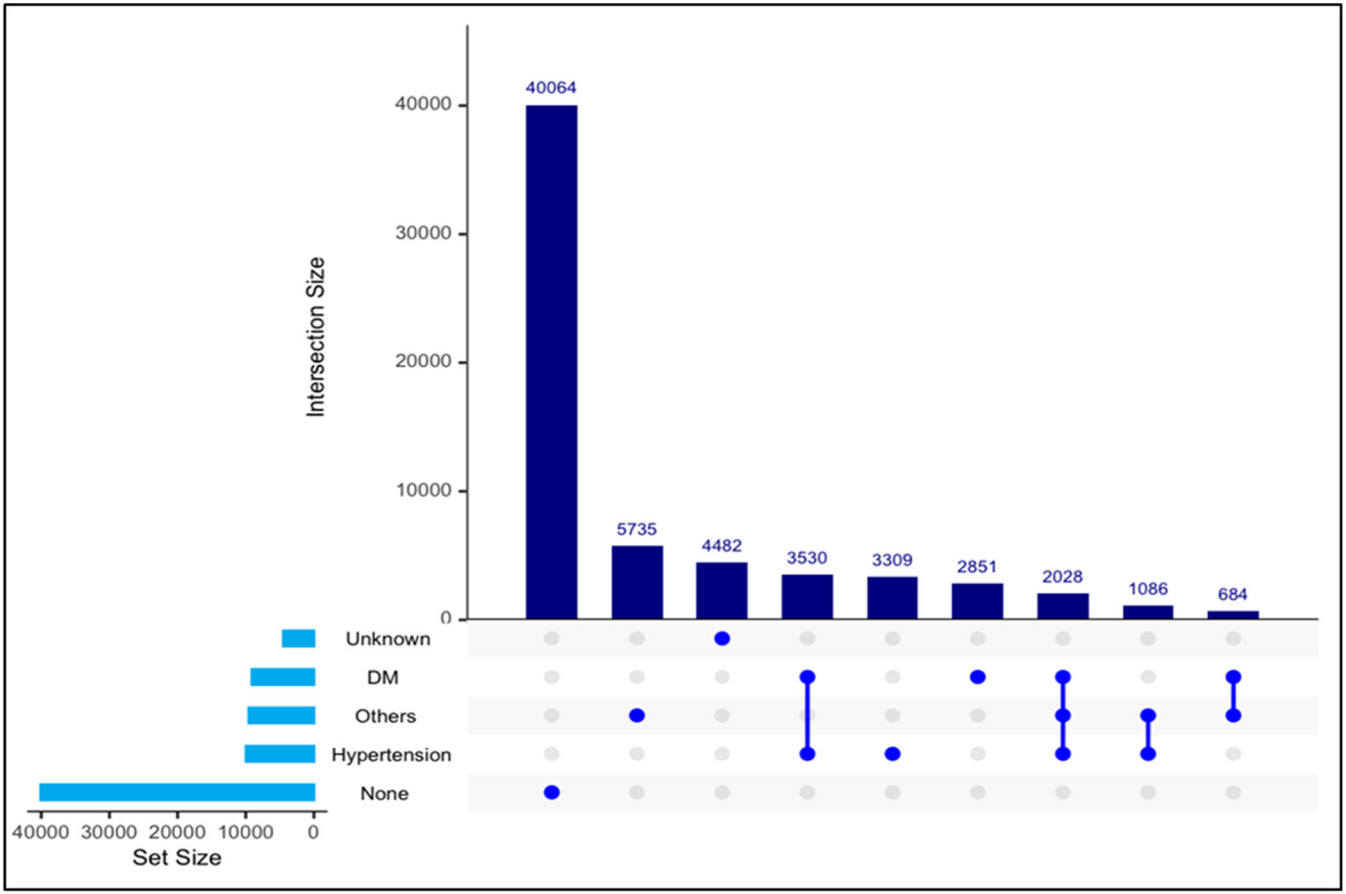
Comorbidities upset plots for combination analysis for patients who have been transported.

**Figure 5:**
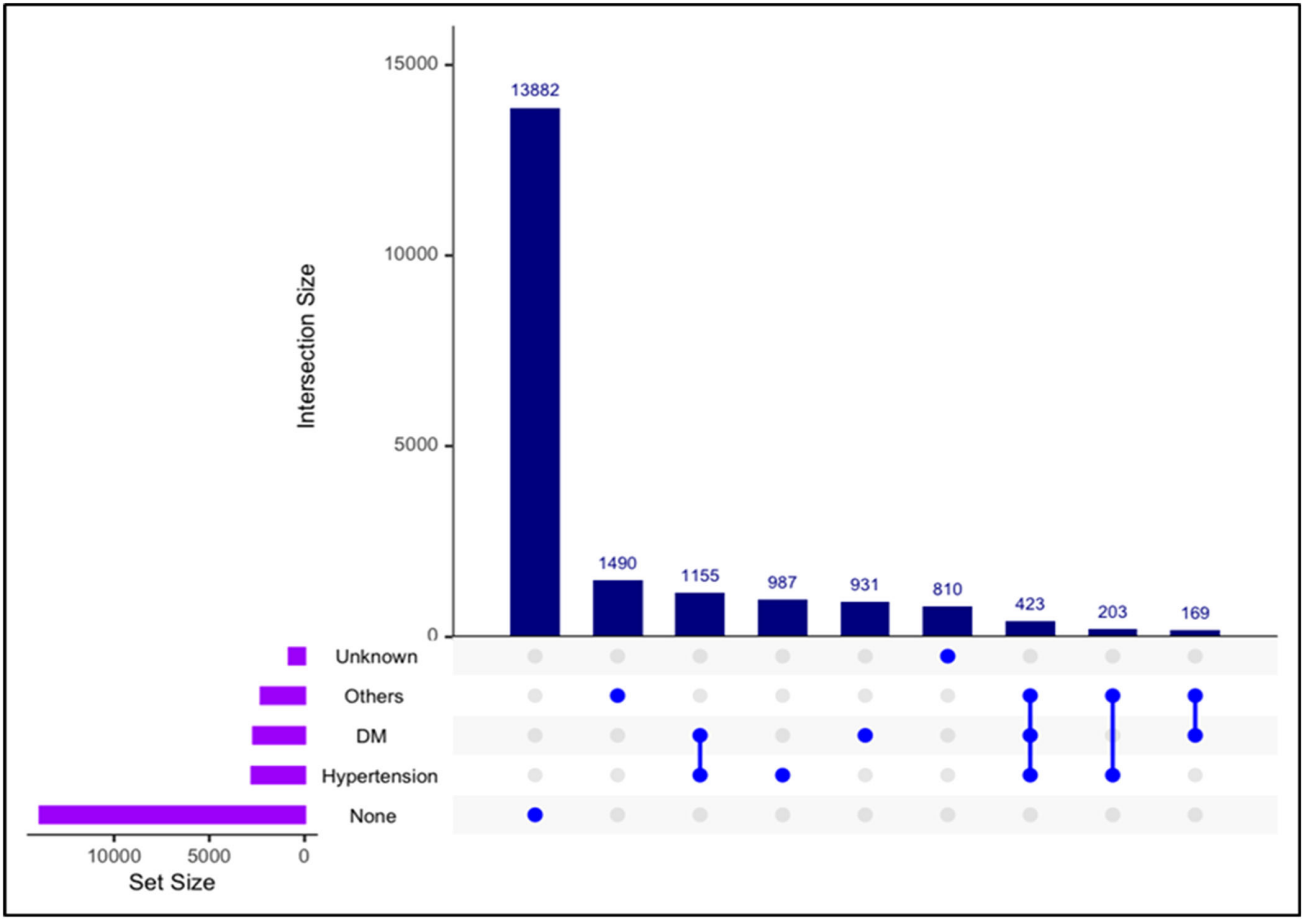
Comorbidities UpSet plots for combination analysis for patients who have not been transported.

UpSet plots, as exemplified in Figures 4 and 5, serve a critical role in the visualization and analysis of complex datasets, particularly when assessing the presence of intersecting data sets. In our study, the UpSet plot was employed to discern the patterns of comorbidities among the patients, specifically the co‐occurrence of DM and hypertension. This type of visual representation is particularly useful for combination analysis as it allows for a clear and concise depiction of how often different conditions appear together within a data set. UpSet plots provide an intuitive means of displaying intersections across categorized groups, such as the coexistence of DM and hypertension among patients. In this case, the plot explained that most patients did not have a significant medical history combining these two conditions regardless of their group categorization. Additionally, Figure 4 offers a comparative insight, highlighting that the comorbidity of DM and hypertension was more frequently observed within the ‘Transported’ group versus the “Not Transported” group, suggesting potential implications for patient transport decisions. UpSet plots helped facilitate a better understanding of the underlying patterns in patient medical histories and their possible impacts on treatment and transport outcomes.

## 4 DISCUSSION

Deciding patient transportation ensures the effectiveness of the 999‐emergency response and mitigates morbidity and mortality risks. This cohort study explored various dimensions of EMS utilization and identified determinants of conveyance decisions, revealing both congruence and departure from the prevailing literature.

One salient finding was the conspicuous male predominance across transported and non‐transported cohorts, which resonates with empirical evidence from recent studies of non‐conveyance decisions in Gulf Cooperation Council (GCC) countries, including during the COVID‐19 pandemic, and highlighted a similar male‐centric inclination for EMS activation.^18^

We identified a marked representation of South Asian demographics, especially within transported patient groups. Juxtaposed against recent literature, as most South Asian populations include low‐income workers compared to patients from other ethnicities, the prehospital healthcare service in Qatar is equally accessible to both citizens and expatriate populations.^6^, ^19^ Broader ethnicity‐in‐healthcare discussions inevitably entangle complex strands of socioeconomic status and health behaviors^20^ that are potentially insightful.

A temporal pattern in our data set indicated a 9 a.m. to 12 p.m. surge in patient‐conveyance activities, potentially indicating workplace‐associated stressors as important triggers, as previously reported.^21^ Such discernments can strategically guide EMS resource allocation and optimize spatiotemporal response protocols.

For locational tendencies, our data unequivocally positions “Homes” at the epicenter of emergencies. Though granular household risk factors as potential emergency catalysts have been investigated, other issues, such as compromised indoor air quality, deficient lighting, and structural integrity of domiciles as risk multipliers, have been highlighted in empirical studies.^22^ Identifying contributory variables necessitates a comprehensive home‐safety exploration model involving community education, audits, and interagency collaboration for safer dwellings. Moreover, the free prehospital healthcare in Qatar potentially incentivises the use of emergency care at home and refusal of transportation, possibly to avoid congested Emergency Departments (ED). Conversely, some, anticipating bypassing extended ED waiting times,^23^ may consent to transportation. Public awareness campaigns could guide the public towards alternative healthcare options, such as health centers while clarifying optimal care pathways.

The unpredictable nature of medical emergencies, as evident from our cohort's considerable representation of patients without known comorbidities, disrupts conventional clinical expectations. Despite the considerable literature on health conditions that amplify the risk of emergencies,^24^ our observations prompt a broader investigation considering, for instance, the elements of medical crises identified by other researchers,^25^ including latent environmental factors, genetic propensities, and undiagnosed medical conditions. During patient assessment, HMCAS clinicians should evaluate the big picture, not just medical history, but also their environmental, genetic, and psychosocial domains. Within this framework, the HMCAS advocates the use of the IMIST‐AMBO (Identification, Medical complaint/Mechanism, Injuries/Information related to the complaint, Symptoms, Treatment, Allergies, Medication, Background history, and other information) during patient handovers within healthcare facilities. IMIST‐AMBO ensures that essential details pertaining to patient complaints are communicated consistently, thus mitigating the risk of oversight and constitute a particularly beneficial approach compared to other handover tools (e.g., Situation, Background, Assessment, Recommendation [SBAR]), which might inadvertently bypass certain contextual and comorbidity‐related details.^26^ Additionally, the HMCAS has institutionalized exemplary EMS standards through the CPGs^8^ that underline the significance of persistent professional upskilling amid inherent uncertainties in the clinical practice.^27^

In our examination of the comorbidities, the emphasis on DM and hypertension aligns seamlessly with current research trajectories^21^ between these morbidities and heightened vulnerabilities that accentuate the urgency for more specialized care protocols and documentation systems for post‐event symptoms.^28^

Bivariate and logistic ridge regression analyses enhanced our understanding of the determinants of the ‘Transported’ versus “Not Transported” outcomes and improved model stability and predictive validity. This multitiered approach provides a nuanced understanding of factors influencing the likelihood of transportation. It yields a model that is particularly effective in predicting which cases are most likely to require transportation, thus offering actionable insights for clinical decision‐making.

The findings explicated by the combination analysis using the UpSet plot carry profound implications for the stratification and management of patient care, particularly in the emergency medical context. It is evident from Figures 4 and 5 that comorbidities such as DM and hypertension are commonly present in conjunction with most patients with comorbidities studied. This observation might suggest recalibrating the clinical assumptions regarding comorbidities within the population. Figures 4 and 5 revealed a marked propensity for the dual presence of these conditions in the ‘Transported’ group, compared to the ‘Not Transported’ cohort. This differential pattern highlights the need for heightened clinical vigilance and resource allocation for transporting patients more likely to present with complex medical backgrounds. Such findings advocate for ‘tailored’ patient assessment protocols, ensuring these comorbidities are considered in therapeutic decision‐making. The utility of UpSet plots enables healthcare decision‐makers with a nuanced understanding of patient comorbidities, guiding more informed and efficacious intervention strategies.

In summary, this study's empirical findings regarding EMS and patient conveyance decisions emphasize the layered complexities that affect conveyance decisions. Nuanced demographic insights into clinical ambivalence demonstrate the intricate prehospital work environment, necessitating sustained academic engagement and introspection for optimized service delivery.

## 5 LIMITATION

Complemented by the ridge regression model, the descriptive data emphasized the crucial role of missing information in clinical examination and model formulation. Despite HMCAS' use of a digital system for recording clinical and nonclinical details, a significant percentage of data was missing, which, if found, could enhance the validity of our conclusions and provide insights that more closely mirror real‐world scenarios. Furthermore, despite careful consideration, strong interrelations between some variables increased the risk of multicollinearity and potential confounders, adding complexity to data interpretation.

Additionally, it is important to acknowledge the inherent limitations associated with the retrospective design of our study. Such a design is prone to risks of errors and biases that are typically less prevalent in prospective studies, such us recall and selection biases, which may influence the generalizability of our findings.

## 6 CONCLUSION

This prehospital study highlights the intricate variables influencing patient‐conveyance decisions, spanning sociocultural factors and clinical ambiguities. Our findings corroborate and challenge the existing literature. Notably, patterns such as male predominance and activity spikes during specific hours necessitate advanced analytical techniques for insightful interpretations. The unpredictable nature of the prehospital setting warrants enhanced training and comprehensive patient assessment approaches that consider factors beyond medical history. Moreover, our methodological challenges emphasize the importance of refining the analytical tools. Our study underscores the dynamic nature of prehospital care and stresses the need for continuous academic engagement. As the prehospital landscape evolves, this study emphasizes the importance of innovation and introspection in successful and effective emergency care delivery.

## AUTHOR CONTRIBUTIONS

**Hassan Farhat**: Conceptualization; investigation; writing—original draft; visualization; formal analysis; data curation; software; methodology; writing—review & editing; funding acquisition. **Guillaume Alinier**: Writing—review & editing; supervision; validation. **Kawther El Aifa**: Writing—review & editing; validation. **Ahmed Makhlouf**: Validation. **Padarath Gangaram**: Conceptualization; project administration. **Ian Howland**: Project administration. **Andre Jones**: Writing—review & editing. **Cyrine Abid**: Writing—review & editing. **Mohamed Chaker Khenissi**: Project administration; resources. **Ian Howard**: Project administration. **Moncef Khadhraoui**: Supervision; project administration. **Nicholas Castle**: Project administration. **Loua Al Shaikh**: Project administration. **James Laughton**: Supervision; Writing—review & editing; resources. **Imed Gargouri**: Project administration; supervision.

## CONFLICT OF INTEREST STATEMENT

The authors declare no conflict of interest.

## ETHICS STATEMENT

The study was conducted in accordance with the guidelines of the Declaration of Helsinki and approved by the Hamad Medical Corporation Medical Research Center (reference MRC‐01‐22‐264).

## TRANSPARENCY STATEMENT

The lead author Hassan FARHAT affirms that this manuscript is an honest, accurate, and transparent account of the study being reported; that no important aspects of the study have been omitted; and that any discrepancies from the study as planned (and, if relevant, registered) have been explained.

## Supporting information

Supporting information.

## Data Availability

Anonymised data that support the findings of this study are available from the corresponding author for review upon request. The data is available from the first author and can be provided upon a reasonable request pending the approval of the ethical board of the medical research centre of Hamad Medical Corporation. The data that support the findings of this study are available on request from the corresponding author. The data are not publicly available due to privacy or ethical restrictions.
