# Supplementary material for: Epidemiology of prehospital emergency calls according to patient transport decision in a middle eastern emergency care environment: Retrospective cohort‐based
Source: Health Sci Rep. 2024 Apr 23;7(4):e2056. doi: 10.1002/hsr2.2056 (PMC11039809; doi:10.1002/hsr2.2056)

**Appendixes**

**Appendix 1: R packages description**

| Package Name | Use Description |
| --- | --- |
| readxl | Reading Excel files. |
| writexl | Writing data to Excel without dependencies. |
| dplyr | Data wrangling and manipulation. |
| forcats | Working with categorical variables. |
| lubridate | Handling and working with date-times. |
| tidyverse | Suite of packages for data manipulation and visualisation. |
| tidyr | Tidying data structures. |
| janitor | Cleaning data and tabulating results. |
| flextable | Formatting tables for reporting. |
| gtsummary | Producing descriptive statistics tables. |
| gt | Creating tables for reporting. |
| mice | Imputation for missing data. |
| skimr | Summary statistics for data inspection. |
| pacman | Package management (loading and checking packages). |
| Maps/ggmap | Drawing geographical maps. |
| qcc | Quality control charts for monitoring processes. |
| ggplot2 | Creating complex visualisations. |
| gridExtra | Arranging multiple grid-based plots. |
| cowplot | Streamlined plot theme and plot annotations for ggplot2. |
| gghighlight | Highlighting points and lines in ggplot2. |
| ggpubr | Adding publication-ready themes to ‘ggplot2’. |
| bdsmatrix | Matrices for mixed-effects models. |
| broom | Converting statistical outputs to tidy data frames. |
| purrr | Functional programming tools. |
| reshape2 | Flexibly reshaping data. |
| rcompanion | Functions to aid statistics students. |
| boot | Bootstrap functions for estimating variance. |
| cramer | Multivariate nonparametric Cramer tests. |
| grid | Functions for drawing grid graphics. |
| vcd | Visualising categorical data. |
| epikit | Tools related to epidemiology. |
| epiR | Epidemiology tools for data and analysis. |
| coin | Coincidence tests for conditional independence. |
| MatchIt | Preprocessing for estimating causal effects. |
| jtools | Analysis and presentation of social science data. |
| car | Extended regression models and tests. |
| MASS | includes many useful functions and data examples, including functions for estimating linear models through generalised least squares (GLS), fitting negative binomial linear models, the robust fitting of linear models, and Kruskal’s non-metric multidimensional scaling.. |

Appendix 2: The pseudocode utilised in this study.

1. START

2. Load necessary libraries:

- tidyverse, readxl, writexl, glmnet, pROC, UpSetR, ggplot2

3. Load Data:

- Read ‘Handover Data.xlsx’ into RawData

4. Preprocess Data:

a. Filter RawData to exclude rows where Handover is NA

b. Create new variables based on existing ones.

c. Handle missing data by imputation or removal.

d. Transform categorical variables into numerical.

e. Divide RawData into transported_data and not_transported_data based on ‘Handover’.

5. Descriptive Analysis:

a. Generate summary statistics, boxplots, and bar plots for selected variables

b. Compute contingency tables

c. Calculate Chi-square test statistics and p-values

d. Create UpSet plots for the combination analysis of comorbidities

6. Numerical Data Analysis:

a. Perform Mann-Whitney U tests on numerical variables

b. Write results to ‘OR for Numerical variables.xlsx’

7. Ridge Logistic Regression Analysis:

a. Examine multicollinearity and remove highly correlated variables

b. Fit logistic regression models using glmnet and perform cross-validation

c. Select the best model using optimal lambda and evaluate model coefficients

d. Create a histogram of predicted probabilities and ROC Curve

8. Visualisation:

a. Generate coefficient plots, ROC curve, histogram of predicted probabilities

b. Create UpSet plots to visualise the comorbidity distributions

9. Output:

a. Write coefficients_df to ‘ridge_regression_coefficients.csv’

b. Generate plots and visualise them to interpret the results

10. END

Appendix 3: Shewhart p-charts of transported patients


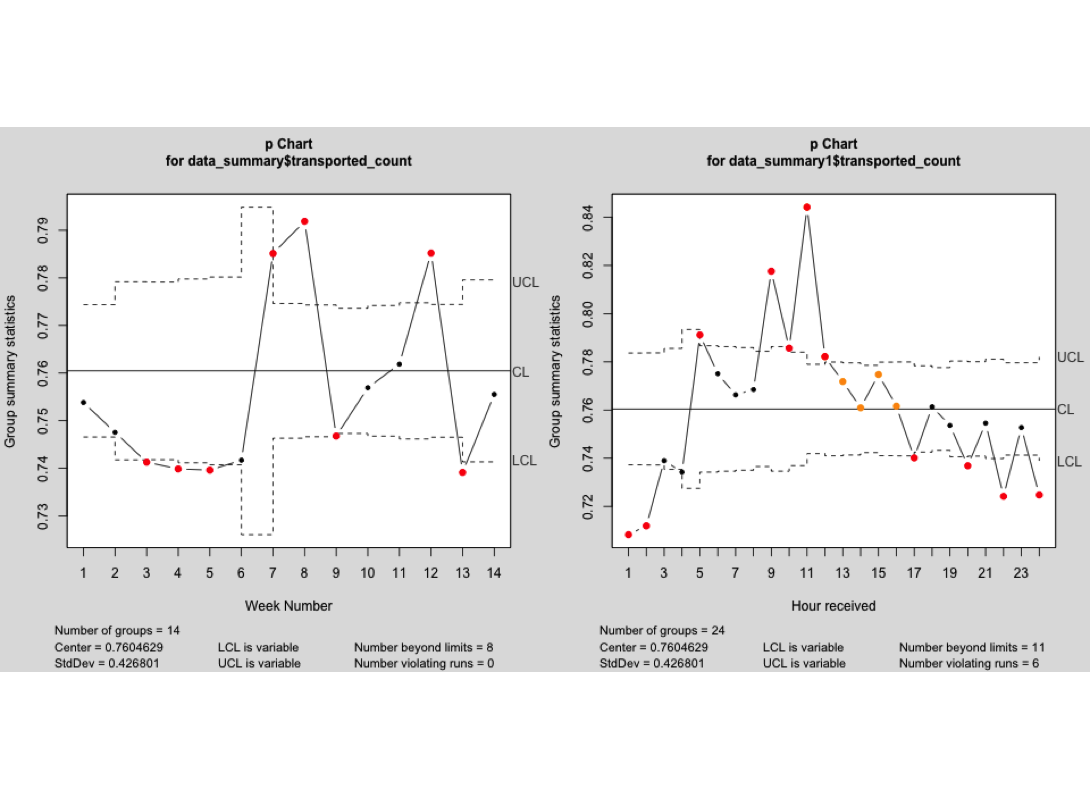


Appendix 4: ProQA call-taking protocol and provisional diagnosis distribution (excluding missing values).


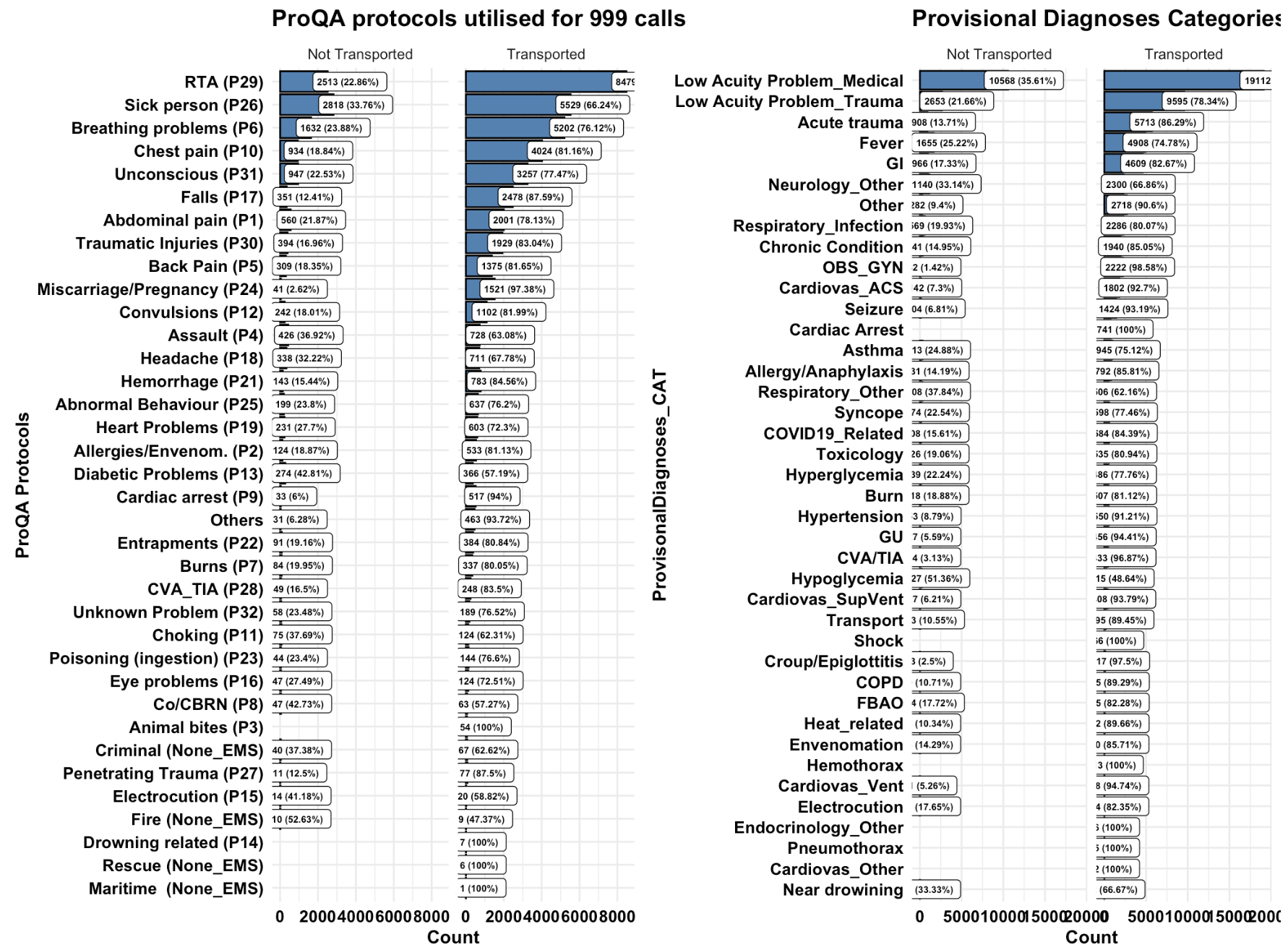

Supplement: Supplementary file 1 — Supporting information. [file HSR2-7-e2056-s001.docx]
